# Complementary and Alternative Medicine on Wikipedia: Opportunities for Improvement

**DOI:** 10.1155/2014/105186

**Published:** 2014-04-17

**Authors:** Malcolm Koo

**Affiliations:** ^1^Department of Medical Research, Dalin Tzu Chi Hospital, Buddhist Tzu Chi Medical Foundation, Chiayi 62247, Taiwan; ^2^Dalla Lana School of Public Health, University of Toronto, Toronto, ON, Canada M5T 3M7

## Abstract

Wikipedia, a free and collaborative Internet encyclopedia, has become one of the most popular sources of free information on the Internet. However, there have been concerns over the quality of online health information, particularly that on complementary and alternative medicine (CAM). This exploratory study aimed to evaluate several page attributes of articles on CAM in the English Wikipedia. A total of 97 articles were analyzed and compared with eight articles of broad categories of therapies in conventional medicine using the Mann-Whitney *U* test. Based on the Wikipedia editorial assessment grading, 4% of the articles attained “good article” status, 34% required considerable editing, and 56% needed substantial improvements in their content. The median daily access of the articles over the previous 90 days was 372 (range: 7–4,214). The median word count was 1840 with a readability of grade 12.7 (range: 9.4–17.7). Medians of word count and citation density of the CAM articles were significantly lower than those in the articles of conventional medicine therapies. In conclusion, despite its limitations, the general public will continue to access health information on Wikipedia. There are opportunities for health professionals to contribute their knowledge and to improve the accuracy and completeness of the CAM articles on Wikipedia.

## 1. Introduction

Wikipedia (http://www.wikipedia.org/), a free and collaborative Internet encyclopedia, has become one of the most popular sources of information available on the Internet since it was launched in 2001. As of November 2013, there were over 4.3 million content articles on the English Wikipedia with over 665 million page edits since Wikipedia was set up [1]. Wikipedia is also the most popular health site on the Internet accessed by unique visitors. Almost 0.4% of the Internet users viewed Wikipedia's medical content on any given day. For example, in the month of October 2013, there were over 171 million hits on 28,020 medical pages on the English Wikipedia [2]. In a search engine-based study, English Wikipedia ranked among the first ten results in 71 to 85% of search engines and health-related keywords tested. In addition, English Wikipedia articles were viewed significantly more often than MedlinePlus Topic [3].

Despite its popularity, there have always been concerns over the quality of health information available on the Internet since its early days [4]. The credibility and quality of information regarding complementary and alternative medicine (CAM) are of no exception [5]. One recent Canadian study assessed the comprehensiveness, reliability, and readability of nephrology articles on Wikipedia and it was concluded that Wikipedia was a comprehensive and fairly reliable medical resource for nephrology patients [6]. Nevertheless, no studies have conducted similar analyses on CAM articles on English Wikipedia. Therefore, the present exploratory study aimed to examine several page attributes including citation characteristics and readability of CAM articles available on the Wikipedia.

## 2. Methods

**Figure 1:**
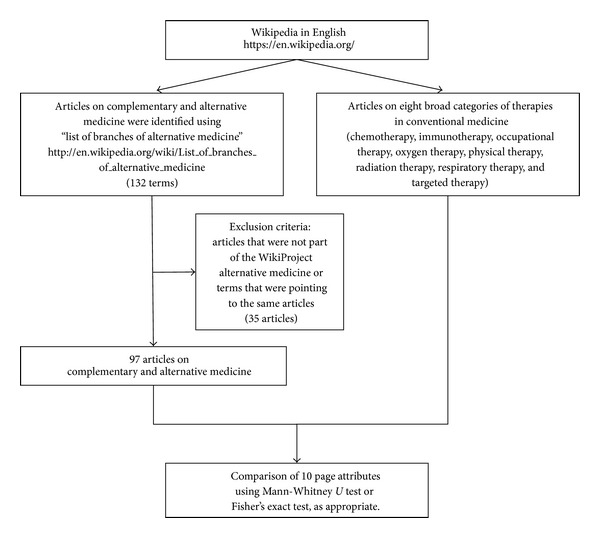
Study flow diagram.

A total of 132 terms included in the “list of branches of alternative medicine” on the English Wikipedia were identified [7] (Figure 1). Terms that were not part of the “WikiProject alternative medicine” [8] or referring to the same article were excluded. The “WikiProject alternative medicine,” as all WikiProjects, was designed primarily to facilitate the development of professional articles on all aspects of CAM. The aim of the WikiProject was to promote the standard of CAM articles by stabilizing controversial topics, developing classification systems, and ensuring the use of high-quality information sources.

After exclusion, 97 remaining articles were analyzed for their page attributes and compared with eight articles of broad categories of therapies in conventional medicine using the nonparametric Mann-Whitney *U* test or Fisher's exact test, as appropriate. The eight articles of therapies in conventional medicine included chemotherapy, immunotherapy, occupational therapy, oxygen therapy, physical therapy, radiation therapy, respiratory therapy, and targeted therapy. Readability was assessed using the simple measure of Gobbledygook (SMOG) [9]. Microsoft Office Excel 2007 (Microsoft Corp., Redmond, WA, USA) was used for data management and IBM SPSS Statistics software package, version 21.0 (IBM Corp., Armonk, NY, USA) was used for data analysis. *P* < 0.05 was considered statistically significant.

## 3. Results

The characteristics of the page attributes of CAM and conventional medicine articles are shown in Table 1. Based on the grading of the Wikipedia editorial assessment [10], 4% of the 97 CAM articles attained “good article” status, 34% required considerable editing, and 56% needed substantial improvements in their content. The median daily access of the CAM articles over the previous 90 days was 372 (range: 7–4,214). The median total edits were 477 (range: 2–10,646) and edits per month ranged from 0 to 87 per article. References with PubMed identifier (PMID) were significantly fewer in CAM articles than those in conventional therapy articles (*P* = 0.015). Regarding citation density, there was a median of 0.8 references with PMID per 1,000 words in CAM articles but 2.9 in conventional medicine articles.

CAM articles were significantly shorter than the conventional therapy articles (*P* = 0.029). A median grade level of 12.7 was required to comprehend the content of the CAM articles. Nevertheless, the SMOG readability was even higher in conventional medicine articles (14.4) (*P* = 0.042).

## 4. Discussion

In this exploratory study of CAM on the English Wikipedia, we reported an overview of the page attributes of 97 CAM articles. In general, there were considerable variations in the page attributes of the articles. First, based on the grading of the Wikipedia editorial assessment, it was found that over half of the articles appeared to be still at their early developing stage and would require further edits to improve their quality. The median daily access of the CAM articles over the previous 90 days spanned from 7 to over four thousands and there was also a wide range in the number of unique editors for each article. There were articles with only two unique editors and therefore greatly varied collaborative efforts were observed depending on the topics of the CAM article. In contrast, the minimum unique editors for conventional therapies article were 64. It is plausible that the quality and reliability of an article are better with diverse contributions by different editors. Future studies should investigate whether this association indeed exists.

Regarding the lower median counts of references with PMID and PMID per 1,000 words in CAM articles compared with conventional therapies articles, two possible explanations could account for this observation. First, some CAM modalities generally have fewer peer-reviewed articles written about them. Second, there are fewer peer-reviewed journals dedicated to publishing CAM studies. As suggested by a study on the quality of Wikipedia on osteosarcoma, more external hyperlinks referring to definitive sources such as those maintained by professional organizations should be included [11].

A significantly higher median SMOG readability score of 14.4 was found in the conventional therapies articles compared with that of CAM articles (12.7). This relatively high SMOG readability score was not a surprise, since Wikipedia pages had previously been noted to be amongst the hardest to read [12]. One possible solution for improving readability of the CAM articles is to include an alternative article of each of the CAM topics, targeted at a lower reading level, on the Simple English Wikipedia (http://simple.wikipedia.org/).

Despite the lack of demonstrated quality, the general public will continue to access health information on the Internet, including that available on the Wikipedia. Therefore, health professionals could contribute their expertise in improving the accuracy and completeness of the CAM articles on Wikipedia. For example, one can add available research studies published in peer-reviewed scientific journals to the “further reading” section of a CAM article. In addition, one can also try to improve the readability of the CAM articles, which should help readers to comprehend the content, thereby enhancing health quality and patient safety. In 2005, an editorial of the Journal Nature has already encouraged researchers to read Wikipedia cautiously and to amend it enthusiastically in order to make Wikipedia a high-quality global resource [13].

Information on the Internet is constantly changing and the content on Wikipedia is of no exception. Therefore, the findings of this study, which were based on a single time point when the study was conducted, might change over time. Nevertheless, the cross-sectional comparisons between CAM articles and conventional therapies articles showed that there were several significant differences in their page attributes. In addition, the choice of the eight broad categories of therapies in conventional medicine might not be able to represent all the articles on conventional therapies and therefore we could not rule out the possibility of selection bias.

In conclusion, this exploratory study of CAM articles available on English Wikipedia showed that there are ample opportunities for health and CAM professionals to contribute their knowledge. By improving the accuracy and completeness of CAM articles on Wikipedia, not only will CAM users be able to find more reliable information from the World Wide Web, but also non-CAM users may find it easier to gain an understanding of the theories and applications of various CAM modalities.

## Figures and Tables

**Table 1:** Characteristics of page attributes of articles on complementary and alternative medicine and on conventional medicine.

Attribute	Frequency or median (min–max)		*P*
	CAM articles (*n* = 97)	Conventional therapy articles (*n* = 8)	
WikiProject quality class			
Featured article & A	0	0	0.042
Good article	4	0	
B & C	33	7	
Start, stub	57	1	
Not available	3	0	
Daily access over the past 90 days	372 (7–4,214)	1,063 (129–3,236)	0.116
Median total edits	477 (2–10,646)	1,105 (138–2,697)	0.293
Edits per month	5 (0–87)	9 (2–21)	0.411
Unique editor	198 (2–2,330)	468 (64–1,296)	0.218
References with PMID	2 (0–130)	10.5 (0–46)	0.015
References with ISBN	2 (0–70)	1 (0–5)	0.212
Word count	1,840 (371–17,197)	5,032 (1,432–8,172)	0.029
PMID per 1000 words	0.8 (0–15.5)	2.9 (0–11.4)	0.056
SMOG readability	12.7 (9.4–17.7)	14.4 (11.6–15.2)	0.042

CAM: complementary and alternative medicine; PMID: PubMed unique identifier; ISBN: international standard book number; SMOG: simple measure of Gobbledygook.
